# Endo‐Epicardial vs. Endocardial‐Only Catheter Ablation of Ventricular Tachycardia in Patients With Ischemic Cardiomyopathy: The EPIC‐VT Trial Design

**DOI:** 10.1111/jce.16630

**Published:** 2025-03-18

**Authors:** Raphaël P. Martins, Pierre Groussin, Francis Bessière, Laure Champ‐Rigot, Jean‐Baptiste Gourraud, Sophie Lepage, Jacques Mansourati, Grégoire Massoulie, Philippe Maury, Sandro Ninni, Bertrand Pierre, Frédéric Sacher, Emilie Varlet, Xavier Waintraub, Clara Locher, Dominique Pavin, Philippe Mabo, Christophe Leclercq, Karim Benali

**Affiliations:** ^1^ Univ Rennes, CHU Rennes, INSERM Rennes France; ^2^ Department of Cardiac Surgery “Louis Pradel” Cardiologic Hospital Lyon France; ^3^ Department of Cardiology University Hospital of Caen Caen France; ^4^ Department of Cardiology CHU Nantes Nantes France; ^5^ Department of Cardiology CHU Angers Angers France; ^6^ Department of Cardiology CHU Brest Brest France; ^7^ CHU Clermont‐Ferrand, Cardiology Department Clermont‐Ferrand France; ^8^ Centre Hospitalier Universitaire de Toulouse Toulouse France; ^9^ CHU Lille, Institut Coeur‐Poumons, Cardiac Intensive Care Unit, Department of Cardiology Lille France; ^10^ Department of Cardiology and Cardiac Surgery Tours University Hospital Tours France; ^11^ Hôpital Cardiologique du Haut‐Lévêque Université Bordeaux II Bordeaux France; ^12^ European Georges Pompidou Hospital, Cardiology Department Paris France; ^13^ Department of Cardiology CHU Pitié Salpêtrière Paris France; ^14^ Saint‐Étienne University Hospital Center, Saint‐Étienne University France

**Keywords:** catheter ablation, Epicardial, ventricular tachycardia

## Abstract

**Introduction:**

Radiofrequency ablation is a cornerstone therapy for patients with ischemic cardiomyopathy (ICM) presenting with ventricular tachycardia (VT). In this context, ablation is typically performed endocardially as a first‐line approach. However, despite acute procedural success, the risk of recurrence remains high, potentially due to the presence of epicardial substrate. Several observational studies have suggested the potential benefits of a first‐line endo‐epicardial approach in decreasing recurrence. In this context, the EPIC‐VT trial was designed to compare endocardial‐only ablation versus combined endo‐epicardial ablation as a first‐line approach in ICM patients with VT.

**Methods:**

The EPIC‐VT trial is a prospective, multicenter, controlled, randomized, open‐label superiority trial with two parallel groups (endocardial‐only approach vs*.* combined endo‐epicardial approach) in a 1:1 ratio. The primary objective of this study is to demonstrate that a combined endo‐epicardial approach reduces the risk of VT recurrence compared to an endocardial approach alone in patients with ICM. Patients will be followed for 2 years after the procedure.

**Results and Conclusion:**

To date, only retrospective studies have compared VA recurrences in patients with ICM, depending on whether ablation was performed using an endocardial or an endo‐epicardial approach, with conflicting results. A meta‐analysis suggested an advantage of the endo‐epicardial approach over the endocardial approach (odds ratio = 0.39 [95% CI: 0.18–0.83]). However, the level of evidence remains low, and no controlled randomized study has confirmed this hypothesis. If the EPIC‐VT study confirms the superiority of a first‐line endo‐epicardial approach, such strategy could become the preferred option for VT ablation in ICM, thereby reducing the risk of VA recurrence.

**Abbreviations**
ATP: antitachycardia pacing
CABG: coronary artery bypass grafting
CRF: case report form
e‐CRF: electronic case report form
ICD: implantable cardiac defibrillator
LV: left ventricle
MRI: magnetic resonance imaging
PFA: pulsed field ablation
VF: ventricular fibrillation
VT: ventricular tachycardia

## 1 Introduction

Patients with ischemic cardiomyopathy (ICM) are at significant risk of ventricular tachycardia (VT), which causes substantial morbidity and mortality [1]. Primary prevention of arrhythmia‐related mortality largely involves implantation of an automatic implantable cardioverter‐defibrillator (ICD) [2]. However, while ICDs effectively terminate arrhythmias, they do not prevent them, and device therapies are associated with considerable morbidity and mortality. Medical therapy using antiarrhythmic drugs (AAD) is a common antiarrhythmic strategy in this population; however, their efficacy remains limited. In cases where AAD are ineffective or poorly tolerated, radiofrequency ablation has emerged as an efficient alternative to reduce arrhythmia recurrences [3]. In the context of ICM, ablation is typically performed using a first‐line endocardial approach, accessing the left ventricle (LV) either retrogradely or transeptally [4]. However, despite achieving acute success during the procedure, the VT recurrence rate remains non‐negligible during follow‐up, with 40%–50% of patients experiencing at least one recurrence within the 2 years after the procedure [5, 6, 7, 8]. These recurrences may be due to an inability to create transmural lesions. In fact, with an endocardial approach, ablation lesions are created only within the LV, whereas a significant portion of the arrhythmogenic substrate may be located on the other side of the myocardial wall, on its epicardial surface [9].

First described in 1996, epicardial ablation has since seen significant developments [10]. The clinical efficacy of epicardial ablation, along with technological advancements and increased operator experience, has allowed this technique to become more widely used, and is now regularly performed in certain clinical situations [11, 12, 13]. Indeed, a dual endo‐epicardial approach is commonly used as first‐line treatment for VT associated with primary dilated cardiomyopathy, myocarditis, or arrhythmogenic right ventricular dysplasia, where the arrhythmogenic substrate is frequently epicardial [11, 12, 13]. A benefit in reducing the risk of recurrence has been demonstrated, with a low complications rate in experienced centers [11].

In contrast, for VT associated with ICM, the combined endo‐epicardial approach is usually reserved for patients with recurrent VT despite one or more endocardial procedures and is not currently considered a first‐line treatment. However, epicardial involvement is often present in ICM VTs [9]. Several retrospective studies [14, 15, 16, 17, 18, 19] and meta‐analyses [20, 21] suggested a significant benefit of the endo‐epicardial approach compared with the endocardial‐only approach. However, the level of evidence remains low, as no randomized trials have confirmed these results.

The aim of our prospective, multicenter, randomized controlled trial is to compare the time to VT recurrence following first‐line ablation in ICM using either an endocardial‐only or a combined endo‐epicardial approach. The findings of this study could establish a new treatment paradigm for managing VA in ICM patients.

## 2 Methods

### 2.1 Study Design

The EPIC‐VT trial is a prospective, multicenter controlled, randomized, open‐label superiority study with two parallel groups, randomized in a 1:1 ratio. The trial includes 13 centers across France (Angers, Bordeaux, Brest, Caen, Clermont‐Ferrand, Lille, Lyon, Nantes, Paris La Pitié Salpêtrière, Paris Hôpital Européen Georges Pompidou, Rennes, Tours). Patients with ICM requiring a first radiofrequency ablation for VT will be randomized to endocardial‐only ablation or combined endo‐epicardial ablation. The study was approved by the Ethics Committees and complies with the principles of the Declaration of Helsinki. Patient recruitment began on October 23, 2023 (first patient recruitment), and is expected to be concluded on October 23, 2028. The duration of study participation for each patient will be up to 24 months from the date of the ablation procedure, while the total study duration will span 60 months.

### 2.2 Patient Population

Participation in the study may be offered to ICM patients requiring VT radiofrequency ablation, either during consultation (outpatient) or during hospitalization (for inpatients requiring urgent intervention). Participation in the study will be proposed and explained to the patient along with the provision of an information letter. Signed informed consent will be obtained before patients are randomized. Antithrombotic treatment will be adapted according to the randomization group. Randomization will be centralized through the Ennov software (Ennov Clinical, Groupe Ennov, Paris, France), generating blocks of variable size to ensure the unpredictability of treatment allocation. The block sizes and order of treatment within each block will be randomly generated. Randomization will be stratified by center.

Patients must meet the following criteria to be eligible for the study: (i) patients aged > 18 years, (ii) requiring first‐time radiofrequency ablation for VT associated with ICM, (iii) implanted with an ICD and monitored remotely through telemonitoring, (iv) for women of childbearing age, use of effective contraception until hospital discharge, (v) provision of written informed consent, and (vi) affiliated with a health insurance system or benefiting from such coverage.

Patients meeting any of the following criteria will be excluded from the study: (i) history of cardiac surgery compromising epicardial access (such as coronary artery bypass grafting, valve replacement, or other surgeries that may have caused pericardial adhesions), (ii) presence of a left ventricular thrombus detected on pre‐procedure imaging, (iii) use of anticoagulant therapy that cannot be temporarily discontinued, (iv) use of dual antiplatelet therapy that cannot be temporarily replaced by single antiplatelet therapy, (v) history of pericarditis, (vi) history of thoracic radiotherapy, (vii) contraindication to general anesthesia, (viii) pregnant or breastfeeding women, (ix) history of type 2 heparin‐induced thrombocytopenia (as heparin injection is required during the procedure), and (x) adults under legal protection (e.g., guardianship, curatorship), deprivation of liberty, or inability to provide informed consent.

### 2.3 Intervention

The aim of this study is to assess whether combined endo‐epicardial radiofrequency ablation is superior to the conventional endocardial approach as a first‐line treatment for ICM patients requiring VT ablation.

#### 2.3.1 Preoperative Assessment

Imaging (such as a cardiac computed tomography (CT) scan with contrast injection, contrast echocardiography, or transesophageal echocardiography) will be performed within the month before the procedure to rule out the presence of an intraventricular thrombus, which would contraindicate endocardial access. If an intraventricular thrombus is detected in a patient not receiving anticoagulant therapy, the procedure will be postponed until the thrombus resolves. A second preoperative imaging will be performed to confirm clearance of the thrombus before proceeding.

Cardiac magnetic resonance imaging (MRI) will not be systematically performed as part of the screening process, as its spatial resolution may be insufficient to definitively exclude epicardial or midmyocardial substrate, and many patients with ICDs may have suboptimal MRI quality or contraindications to MRI. However, in cases where MRI was performed before the procedure, a secondary analysis will be conducted to evaluate its predictive value in identifying epicardial or midmyocardial substrate.

Before the procedure, a clinical examination and standard preoperative blood test will be performed. The procedure will be performed under local anesthesia with light sedation, if needed, or under general anesthesia, depending on the patient's preference and logistical considerations at each center.

#### 2.3.2 Endocardial Approach Only

Regarding antithrombotic management: (i) for patients on direct oral anticoagulants (DOACs), the morning dose (apixaban, dabigatran, or morning dose of rivaroxaban) or the evening dose (evening dose of rivaroxaban) will be withheld; (ii) for patients on vitamin K antagonists (VKAs), the procedure will be performed if the international normalized ratio (INR) is < 3 on the day before the procedure; and (iii) for patients on antiplatelet agents, the procedure will be performed regardless of the combination of antiplatelet therapy.

The defibrillator will be deactivated at the beginning of the procedure. Endocardial access to the left ventricle will be obtained via a retrograde aortic approach (through femoral arterial puncture) and/or a transseptal approach (through femoral venous puncture) according to the center's usual practice and the presumed location of the VT. A quadripolar catheter will be placed inside the right ventricle to pace the ventricle during the procedure if needed for VT induction/overdrive and to perform programmed ventricular stimulation at the end of the procedure.

Unfractionated heparin will be administered at the beginning of the procedure (100–120 IU/kg) and then regularly during the procedure to maintain an activated clotting time (ACT) of approximately 300 s.

The procedure will be performed using conventional 3D mapping techniques. A high‐density map during sinus rhythm or pacing will be created at the beginning of the procedure to identify low‐voltage areas and the presence of late potentials, abnormal ventricular electrograms, and deceleration zones. VT induction will be attempted if desired to perform an activation map. A complete substrate ablation strategy will be employed, supplemented by specific strategies for the ablation of induced VTs (pace mapping and entrainment mapping). Radiofrequency energy will be delivered using irrigated tip catheters at 30–50 W, preferably with isotonic saline, for a maximum duration of 60 s.

At the end of the procedure, programmed ventricular stimulation will be used to assess the inducibility of clinical or other nonclinical VTs, thereby determining the acute success or failure of the procedure. A standardized protocol will be followed, with stimulation at two different cycle lengths (600 and 400 ms) and up to three extrastimuli decrementing to 200 ms (or the ventricular refractory period). For non‐inducible patients, the procedure will be concluded. For patients with inducible VTs, ablation will continue, specifically targeting the induced VTs.

Acute procedural success will be defined by both the absence of VT inducibility on programmed ventricular stimulation and complete substrate modification, including the elimination of arrhythmogenic sites such as local abnormal ventricular activities. To confirm the efficacy of substrate modification, systematic postablation remapping will be performed.

Crossover to an epicardial approach will not permitted during the index procedure. If VT remains inducible despite endocardial ablation, patients will continue follow‐up as per protocol. Any recurrence of VT will be considered as meeting the primary outcome, and subsequent management, including potential epicardial access, will be left to the discretion of the treating physician outside of the initial study intervention. Patients will be analyzed on an intention‐to‐treat basis.

The defibrillator will be reactivated at the end of the procedure.

#### 2.3.3 Combined Endo‐Epicardial Approach

Regarding antithrombotic management: (i) for patients on DOACs, the last dose should be taken at least 3 days before the procedure (apixaban and rivaroxaban) or 4 days before (dabigatran); (ii) for patients on VKAs, therapy will be discontinued 5 days before the procedure, which can be performed if the INR is < 1.5 on the day before the procedure; (iii) for patients on antiplatelet agents, aspirin will be continued alone and any other antiplatelet agent will be halted at least 5 days before the procedure.

The defibrillator will be deactivated at the beginning of the procedure. A quadripolar catheter will be placed inside the right ventricle to pace the ventricle during the procedure if needed for VT induction/overdrive and to perform programmed ventricular stimulation at the end of the procedure.

The first step of the procedure will aim at obtaining pericardial access, which will be performed using a Tuohy needle. To minimize the risk of complications, an anterior approach will be preferred, using fluoroscopy in the lateral projection to avoid diaphragmatic puncture, and a long guidewire will be placed before advancing the sheath to confirm intrapericardial placement. Other specific techniques may be used according to the operator's preference (e.g., micropuncture, CO2 insufflation). A deflectable sheath will then be introduced into the pericardium, and a high‐density mapping of the epicardium in sinus rhythm/pacing will be performed to identify low‐voltage areas and search for the presence of late potentials, abnormal ventricular electrograms, and deceleration zones. The left phrenic nerve will be located by high‐output stimulation (10 mA), and its position annotated on the map.

After completing the epicardial map, before mapping the left ventricular endocardium, and in the absence of pericardial bleeding related to the puncture, unfractionated heparin will be administered (100–120 IU/kg) and then regularly during the procedure to maintain an ACT of approximately 300 s.

Endocardial access to the left ventricle will be obtained via a retrograde aortic approach (through femoral arterial puncture) and/or a transseptal approach (through femoral venous puncture) according to the center's usual practice and the presumed location of the VT. A high‐density map during sinus rhythm or pacing will be created to identify low‐voltage areas and the presence of late potentials, abnormal ventricular electrograms, and deceleration zones.

Once the endocardial and epicardial surfaces will have been mapped, VT induction will be attempted if desired to perform an activation map. A complete substrate ablation strategy will be employed, complemented by specific strategies for the ablation of induced VTs (pace mapping and entrainment mapping). Radiofrequency energy will be delivered using irrigated tip catheters at 30–50 W, preferably with isotonic saline, for a maximum duration of 60 s: (i) For the endocardial substrate and VTs, a complete substrate ablation strategy will be employed, complemented by specific strategies for the ablation of induced VTs (pace mapping and entrainment mapping). (ii) For the epicardial substrate and VTs, after identifying the areas requiring ablation (substrate ablation strategy, complemented by specific strategies for the ablation of induced VTs using pace‐mapping and entrainment mapping), their positions relative to the phrenic nerve (previously located by stimulation during mapping) and coronary arteries (using fused imaging from a CT scan or intra‐procedural coronary angiography) will be evaluated to determine whether ablation is achievable.

At the end of the procedure, programmed ventricular stimulation will be performed to assess the inducibility of the clinical or other nonclinical VTs, thereby determining the acute success or failure of the procedure. A standardized protocol will be followed, with stimulation at two different cycle lengths (600 and 400 ms) and up to three extrastimuli decrementing to 200 ms (or the ventricular refractory period). For non‐inducible patients, the procedure will be concluded. For patients with inducible VTs, ablation will continue, specifically targeting the induced VTs.

Acute procedural success will be defined by both the absence of VT inducibility on programmed ventricular stimulation and complete substrate modification, including the elimination of arrhythmogenic sites such as local abnormal ventricular activities. To confirm the efficacy of substrate modification, systematic postablation remapping will be performed.

A pericardial drain may be left in place overnight, and intrapericardial injection steroid injection performed at the discretion of the operator.

The defibrillator will be reactivated at the end of the procedure.

#### 2.3.4 Before Hospital Discharge

Transthoracic echocardiography will be performed on the day after the procedure to ensure the absence of pericardial complications. ICDs will be programmed as recommended by guidelines [22], as follows: (i) VF Zone: ≥ 220 bpm, with high‐energy shocks; program 1 burst of ATP before or during charging; long detection intervals (at least 20) should be programmed; (ii) VT Zone: 10–20 bpm slower than the clinical VT rate if known, potentially separated into VT1 and VT2 zones; at least 3 bursts of ATP before delivering shocks in the VT1 zone, and at least 1 burst in the VT2 zone, followed by high‐energy shocks; long detection intervals should be programmed; (iii) Monitor Zone: recommended at 150–160 bpm (if the VT zone extends below this rate, a Monitor Zone is not necessary); (iv) Base Rate: set at 30–40 bpm unless pacing is indicated; (v) SVT discriminators should be programmed ON. Patients will be discharged 24–48 h after the procedure, provided that there are no complications. Upon discharge, a remote‐monitoring report will be scheduled on a monthly basis.

Management of antiarrhythmic drug therapy postablation will be left to the discretion of the treating physician. However, the study protocol recommends discontinuation of AADs whenever feasible. The use of AADs, including initiation, continuation, or discontinuation, will be systematically recorded during follow‐up to assess their potential impact on study outcomes.

### 2.4 Follow‐Up Visit

The patient will receive a telephone call at 6 and 18 months, and will be seen in a consultation at 1 and 2 years following the procedure. This will allow the medical team to inquire about any hospitalizations and the use of antiarrhythmic medications. Remote monitoring will be used to retrieve any recorded arrhythmic event. In the event of hospitalization or consultation, the center or healthcare professional who manages the patient will be contacted to obtain hospitalization reports and check for the occurrence of ventricular arrhythmia.

After 2 years of follow‐up, patients included in the study with a successful procedure and no recurrence will be followed up remotely until (i) arrhythmia recurrence or at the latest, or (ii) the date of the last visit of the last patient included.

Of note, if a new ablation is required during follow‐up due to arrhythmia recurrence, the choice of approach (endocardial only or combined endo‐epicardial approach) will be left to the discretion of the medical team in charge of the patient.

### 2.5 Data Collection and Management

#### 2.5.1 Data Collection

The measurement time points for each assessed parameter are shown in Table 1. Descriptive data for the study population will be collected before the ablation procedure. These data will be presented using descriptive statistics.

**Table 1:** EPIC‐VT protocol.

	Inclusion visit		Randomization	D‐1		Radiofrequency Ablation	Before hospital discharge		6‐month follow‐up phone call^a^ (M6 ± 1 month)		12‐Month consultation (M12 ± 1 month)		18‐month follow‐up phone call^a^ (M18 ± 1 month)		24‐Month consultation (M24 ± 1 month)		Data collection post 24 months	
Actions	CP	AR		CP	AR		CP	AR	CP	AR	CP	AR	CP	AR	CP	AR	CP	AR
Information and Consent		X																
Verification of Selection Criteria		X																
Pregnancy Test		X																
Verification of Medical History	X																	
Standard Preoperative Biological Assessment				X														
Echocardiography							X											
Clinical Examination	X			X			X											
ICD Interrogation							X											
Medications Taken	X			X			X			X		X		X		X		
Imaging (Exclusion of Thrombus)				X^b^														
Inquiry About Adverse Events							X			X								
Remote monitoring (VT/VF episodes)										X		X		X		X		X

Abbreviations: CP = clinical practices, AD = added by the Research.

^a^ or during a scheduled consultation;

^b^ From 1 Month to 1 Day Before.

#### 2.5.2 Data Management

All information required by the protocol will be recorded in electronic case report forms (eCRFs). eCRFs will be completed using an Electronic Data Capture system. Designated, trained staff at each study site will be responsible for data entry into the eCRF and the correction of such data when necessary. In the event of discrepant data, the study sponsor will request data clarification from the sites. The eCRFs will be reviewed, electronically signed, and dated by the investigator or a designee. Missing data will not be replaced.

### 2.6 Outcomes

#### 2.6.1 Primary Outcome

The primary endpoint is survival without ventricular arrhythmia recurrence, defined as the interval between the date of ablation and the date of the first recurrence of ventricular arrhythmia. Ventricular arrhythmia recurrence will be defined as the occurrence of an appropriate therapy delivered by the ICD or the occurrence of sustained VT/VF lasting more than 30 s requiring hospitalization. The occurrence and date of the event will be obtained from ICD interrogation. Patients without recurrence will be censored on the date of the last ICD interrogation.

#### 2.6.2 Secondary Outcome

The secondary outcomes are: (i) the number of ventricular arrhythmias requiring ICD treatment or hospitalization; (ii) the percentage of patients with ventricular arrhythmia recurrence at 2 years; (iii) the percentage of patients experiencing an electrical storm at 2 years, defined as the occurrence of at least three appropriate therapies (ATP or shocks) delivered by the ICD within 24 h; (iv) the rate of serious complications related to the procedure at 1 month; (v) procedure duration (from puncture to catheter removal, in minutes) and radiofrequency duration (in minutes); (vi) the number of patients hospitalized for cardiovascular reasons (e.g., heart failure, arrhythmias) at 2 years; (vii) the number of patients requiring a repeat ablation; (viii) the mortality rate at 2 years; (ix) the number of patients with non‐inducibility at the end of the procedure; (x) the percentage of patients who experienced inappropriate therapy at 2 years; (x) the duration of hospitalization postablation (days from ablation to hospital discharge) to assess post‐procedural recovery.

### 2.7 Sample Size and Statistical Considerations

#### 2.7.1 Sample Size Estimation

In two meta‐analyses investigating ICM patients outcomes after endo‐epicardial ablation, the risk of ventricular arrhythmia recurrence at 2 years was 50% in the ‘endocardial‐only’ group and 26% in the ‘endo‐epicardial’ group (HR = 0.43) [20, 21]. To demonstrate this difference, it is necessary to observe 59 events to ensure 90% power with a two‐sided α risk of 5%. To observe a minimum of 59 events required for analysis, we planned to evaluate 150 patients followed for a period of at least 2 years. After the 2‐year follow‐up, patients will no longer be monitored as part of the study, but the ICDs will be remotely interrogated via telemonitoring for patients with no recurrence until (i) arrhythmia recurrence or at the latest (ii) the date of the last visit of the last patient included.

Of note, the sample size estimation is based on treatment effect derived from a prior meta‐analysis of observational studies. However, we acknowledge that the endocardial ablation strategies varied significantly across the included studies, ranging from targeted ablation of clinical/induced VT to complete scar homogenization. This heterogeneity may have influenced the estimated benefit of a combined endo‐epicardial approach and represents a limitation of the current power calculation.

#### 2.7.2 Statistical Analysis

Statistical analyses will be performed using the biometry unit of the Center of Clinical Investigation of Rennes. Interim analyses will not be performed. Patients randomized and undergoing ablation will be analyzed on a “modified intention‐to‐treat” basis. Only patients with a persistent left intraventricular thrombus that definitely contraindicates ablation will be excluded. The expected exclusion rate is very low ( < 3%) and exclusion is not expected to be dependent on the allocated treatment arm. The clinicopathological criteria at inclusion will be described for all patients as well as for each of the two study arms: (i) qualitative variables will be presented as counts and percentages, and (ii) quantitative variables will be presented as means, standard deviations, quartiles, minimums, and maximums. The normality of the distribution of quantitative variables will also be assessed graphically and/or using the Shapiro‐Wilk test, if necessary. The primary endpoint is a censored variable; therefore, both groups will be compared using the log‐rank test. Secondary endpoints will be analyzed according to the type of variable: (i) quantitative variables will be analyzed using the Student's *t*‐test or the Mann–Whitney test if necessary; (ii) qualitative variables will be analyzed using the Chi‐square test or Fisher's exact test if necessary; (iii) censored variables will be analyzed using the Log‐rank test.

Adjustments may be made in cases with heterogeneity at inclusion. The conditions for applying these tests or models will be verified before their implementation. All the tests will be conducted at a significance level of 5%.

### 2.8 Limitations

An inherent limitation of this study is the exclusion of patients with prior coronary artery bypass grafting (CABG), a significant subgroup of patients with ischemic cardiomyopathy undergoing catheter ablation. While the inclusion of this group would introduce complexities, particularly in relation to epicardial access, we intentionally excluded these patients to maintain study feasibility and safety. As a result, the findings of this study may not be directly applicable to this subgroup, and further studies specifically focused on patients with prior CABG will be necessary to assess the optimal ablation strategy in this population.

This study exclusively utilizes radiofrequency ablation, and alternative energy sources such as pulsed field ablation (PFA) or large‐footprint radiofrequency catheters are not included. While these technologies are emerging, they are not yet widely implemented for ventricular tachycardia ablation. Given that patient enrollment is already well underway, we anticipate completing the study before the widespread adoption of PFA for ventricular applications. However, the potential future impact of these evolving technologies on VT ablation strategies should be considered when interpreting the generalizability of our results.

## 3 Discussion

Since its first description in 1996 by Sosa et al. [10] epicardial ablation has been increasingly used for catheter ablation of VT. Since then, some cardiomyopathies have been described as having nearly exclusively an epicardial substrate, like Chagas cardiomyopathies or myocarditis, warranting a first‐line epicardial procedure. Conversely, the high success rate of surgical subendocardial resection, as of catheter ablation in patients with ICM [22] has paved the way for a sole endocardial procedure as first line therapy for such patients. However, despite major technological improvements in mapping catheters, ablation tools, and 3D mapping systems, and despite a relatively high acute success rate, many ICM patients will experience a VT recurrence over follow‐up [5, 6, 7, 8, 23]. Many reasons may explain recurrences, such as incomplete endocardial ablation, substrate remodeling [24, 25], or the presence of a hidden substrate in the epicardial face of the ventricle, or buried in the mid‐myocardium. Tung et al elegantly demonstrated that the epicardium was a functional component of the VT circuit in the majority of post‐infarct arrhythmias [9]. Indeed, only 7% of ICM VTs exhibited propagation with complete reentry confined to the endocardium or the epicardium, while the vast majority (73%) had 3D activation patterns across endocardial and epicardial layers.

Consequently, few studies have attempted to evaluate whether a combined endo‐epicardial ablation could result in better procedural outcomes after RF ablation. Some retrospective observational studies have analyzed the recurrence of VT in patients with ICM based on whether the ablation was performed via the endocardial or endo‐epicardial approach, with conflicting results [14, 15, 16, 17, 18, 19]. A recent meta‐analysis of six studies suggests an advantage of the endo‐epicardial approach over the endocardial approach (Relative risk reduction (RRR) = 0.43 [95% CI: 0.28–0.67]). No differences in terms of acute procedural success nor all‐cause mortality were observed, while a nonsignificant trend towards a higher risk of complication was noted (RRR = 1.88 [95% CI: 0.19–18.76]). [20] Bisceglia et al recently reported a low 3.6% risk of complications when epicardial ablation is performed in experienced centers [11]. More recently, Mohanty and al observed in a study of 361 patients with ICM VT that those receiving combined endo‐epicardial ablation had significantly lower 5‐year VT recurrence rates (45.6%) than those with only endocardial ablation (89.1%; *p* = 0.01) [26]. After adjustment, epicardial ablation significantly reduced VT recurrence risk (Hazard Ratio = 0.48; 95% CI = 0.27–0.86; *p* = 0.02). Although many studies have reported the safety and efficacy of a combined strategy in ICM patients, the level of evidence remains low, mainly based on retrospective observations with small patient populations, and no controlled, randomized study has confirmed this hypothesis. Notably, only one clinical trial (NCT02358746) has been registered since 2015 to evaluate this question, but was stopped due to inclusion issues.

The aim of the proposed multicentre, prospective, randomized trial is then to determine whether a first‐line combined endo‐epicardial ablation improves long‐term arrhythmia‐free survival in ICM patients compared to an endocardial‐only approach.

## Ethics Statement

The study has been approved by the National Ethics Committees (CPPs).

## Consent

All patients will give their written informed consent before taking part in the study. Each patient taking part in the study will also provide their written consent for access to their individual data for quality control purposes and the use of their data for the study analysis.

## Conflicts of Interest

The authors declare no conflicts of interest.

## Data Availability

The data that support the findings of this study are available from the corresponding author upon reasonable request.
